# Prolonged Response to Pembrolizumab in Spindle Cell Squamous Cell Carcinoma Metastatic to the Central Nervous System

**DOI:** 10.1177/2324709619850216

**Published:** 2019-05-27

**Authors:** Yuxin Liu, Bailey Fitzgerald, Edward Perry, Ashutosh Pathak, Herta H. Chao

**Affiliations:** 1 Yale University, New Haven, CT, USA; 2 Veterans Affairs Connecticut Healthcare System, West Haven, CT, USA

**Keywords:** metastatic cutaneous squamous cell carcinoma, spindle cell, brain metastases, pembrolizumab

## Abstract

*Background.* Cutaneous squamous cell carcinoma is a common type of skin cancer, with aggressive metastatic or locally advanced disease representing an uncommon minority of presentations. Emerging data have supported the Food and Drug Administration approval of the anti-PD1 human monoclonal antibody cemiplimab in select patients with advanced disease. However, there is limited data regarding durability of effect and generalizability of anti-PD1 effectiveness across therapies. Additionally, information regarding applicability of these regimens to the rare spindle cell variant and to central nervous system metastases for cutaneous squamous cell carcinoma is unfortunately limited. *Case Presentation*. A 72-year-old gentleman presented with facial neurological deficits and a dermal nodule and was diagnosed with spindle cell squamous cell carcinoma with perineural invasion. His course was notable for early intracranial metastasis with progressive neurological deficits despite recurrent radiation therapy with intermittent response. When progressive left-sided weakness prompted imaging evaluation that was concerning for disease recurrence after exhaustion of radiation therapy options, the patient was started on systemic therapy with the anti-PD-1 monoclonal antibody treatment prior to the approval of cemiplimab. Pembrolizumab was chosen due to the fact that the patient was ineligible for clinical trials and for its every 21-day dosing. With this treatment, he has achieved a durable clinical response, resulting in near resolution of neurological deficits and more than a year of progression-free survival to date, despite aggressive intracranial disease. *Conclusions.* This case suggests that anti-PD-1 therapy with pembrolizumab may represent an effective and well-tolerated treatment for patients with metastatic spindle cell squamous cell carcinoma including patients with metastatic disease to the central nervous system.

## Background

Cutaneous squamous cell carcinoma (SCC) is the second most common type of skin cancer with an estimated annual incidence of more than 700 000.^1-3^ Studies have found between 1.9% and 5.2% of SCC metastasize.^4,5^ Risk factors for metastasis include thickness greater than 2.0 cm, poorly differentiated histology, perineural invasion (PNI), and immunosuppression.^4,6-8^ Spindle cell or sarcomatoid SCC is an uncommon variant with poorly differentiated pathology and occurs in areas of the body that receive high degrees of sun damage or have prior radiation exposure.^9-11^ These spindle cell squamous cell carcinomas (SCSCC) present as raised or exophytic nodules that are clinically difficult to distinguish from scar or other types of skin cancer.^12^ Given the rarity of these tumors, literature is sparse with regard to the metastatic potential or prognosis of these lesions.

Although cure rates are high with local disease, the mortality rate from metastatic cutaneous SCC is about 70%.^3^ The treatment paradigms for local disease follow those of other squamous cell cancers including resection and consideration of adjuvant field radiation, but little guidance is available for providers in treating nonresectable or metastatic disease. Pembrolizumab is an immunoglobulin G4 antibody that acts as a checkpoint inhibitor to programmed death receptor 1 (PD-1), which promotes T-cell activation and facilitates antitumor activity. Currently, pembrolizumab has been approved for various malignancies, including melanoma and non–small cell lung cancer, with more clinical trials in other cancers underway.^13^ On September 28, 2018, the Food and Drug Administration has approved anti-PD-1 antibody cemiplimab for the treatment of metastatic or locally advanced cutaneous SCC, following encouraging expansion trials.^14,15^ However, there are limited data regarding durability of effect and generalizability of response to other anti-PD-1 therapies. In this article, we present a case of SCSCC metastatic to the brainstem with favorable response for more than 18 months to anti-PD-1 therapy with pembrolizumab.

## Case Presentation

In 2013, a 72-year-old Caucasian male patient with extensive history of sun exposure presented with right eye pain and associated forehead dysesthesias. He was noted on examination to have a palpable 3 mm dermal nodule within the right lateral eyebrow. Biopsy revealed keratin-positive SCSCC with PNI. Staging computed tomography scans revealed no evidence of metastasis. Mohs surgery performed in February 2014 confirmed a stage 1 lesion without extension to the epidermis and negative surgical margins.

In August 2014, he developed double vision and right upper facial pain. He was found to have a right cranial nerve (CN) VI palsy and partial CN III palsy. The etiology of the right facial pain was not clear at the time. Magnetic resonance imaging (MRI) of brain and computed tomography imaging in September 2014 were negative; however, his symptoms progressively worsened. Repeat MRI of brain in February of 2015 revealed a new 0.6 × 0.5 cm right Meckel’s cave lesion. Due to the location and the size of his central nervous system (CNS) lesion, it was not deemed safe for biopsy by the neurosurgical team. Given the anatomical distribution and symptoms reported by the patient, it was assumed that the SCSCC previously resected from the right eyebrow had tracked along the VI branch of CN V through the cavernous sinus to the right Meckel’s cave resulting in additional cranial neuropathies of CN III and CN VI. The workup for other malignancies was negative. The patient received external beam radiation to the area of the original SCSCC and brain. The radiation resulted in significant improvement in the right upper facial pain. In February 2016, he developed left arm weakness and underwent another surveillance MRI of brain that showed a new extensive T2/FLAIR hyperintensity centered in the right brainstem with a 1.2 cm enhancing lesion in the right pons. He underwent gamma knife therapy that was completed in March 2016 with no recurrence of disease through June 2016.

**Figure 1.**
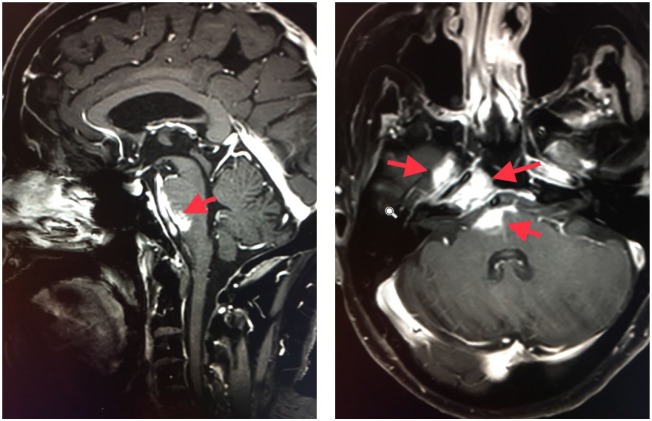
Magnetic resonance imaging (MRI) of metastatic spindle cell squamous cell carcinomas involvement of the brainstem prior to pembrolizumab. Sagittal and axial T1 weighted post-contrast fat-saturated images, performed on Siemens MRI scanner, show intracranial extension of the mass lesion through perineural spread. There is involvement of the right trigeminal nerve along its course through the foramen ovale and Meckel’s cave, which extends posteriorly to involve the anterior surface of pons and medulla on the right side. The axial image also demonstrates the spread along the right abducens nerve along the Dorello’s canal with its cisternal portion extending posteriorly to involve the anterior pontomedullary junction. Enhancing mass lesion is seen along the clivus on the right side as well.

However, in September 2016, he developed recurrent left upper and new lower sided weakness and gait instability. Physical and occupational therapy evaluations at the time showed profound left-sided leg weakness and foot drop requiring bracing and a cane for ambulation. A repeat MRI revealed changes assumed to be radiation-associated necrosis, and he was treated with pulse dose steroids. In January 2017, he was admitted for profound weakness, despite MRI showing stable disease. In May 2017, he presented with vertigo and left eye abduction deficits and worsening left-sided weakness. An MRI showed interval increase in the enhancement of the V3 portion of the right trigeminal nerve extending into the foramen ovale and destruction of the clivus on the right side with involvement of the right sixth CN (Figure 1).

**Figure 2.**
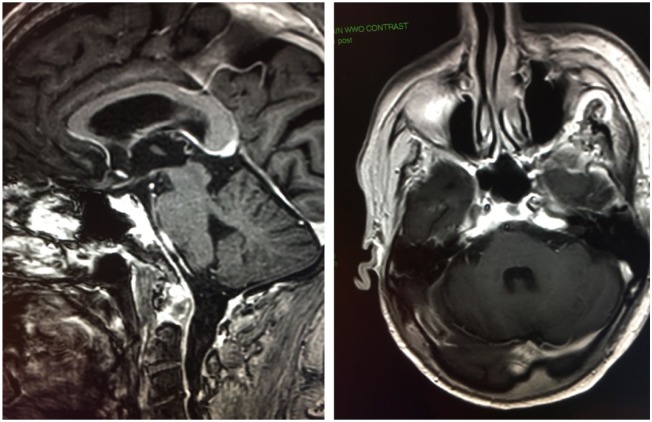
Magnetic resonance imaging of the brainstem following 15 months of pembrolizumab treatment. Comparable axial and sagittal post-contrast fat-saturated images to Figure 1 following treatment demonstrate significant interval improvement with near complete resolution of enhancement along the anterior surface of brainstem. The degree of involvement of the right trigeminal and abducens nerve in the region of Meckel’s cave and Dorello’s canal also shows significant interval improvement.

At this point, the patient was no longer a candidate for any further radiation treatments given extensive prior treatment. He was considered for the SWOG S1609 DART trial (Dual Anti-CTLA-4 and Anti-PD-1 Blockade in Rare Tumors), but the risks of acquiring a biopsy for study enrollment from the brainstem lesion were felt to be too great. The tumor specimen originally resected from the right eyebrow in 2013 was sent for further profiling and found to have retained expression of PMS-2, MLH-1, MSH-6, and MSH-2 PDL1, therefore was unlikely to be microsatellite instability high. However, the PDL1 score was found to be between 1% and 5%. In July 2017, the patient was started on pembrolizumab 200 mg every 3 weeks after his steroid dosing was steadily lowered to prednisone 10 mg. Given the convenience of every 21-day dosing, pembrolizumab was chosen over nivolumab. Within 6 weeks of starting PD1-inhibitors, the patient experienced dramatic neurological improvement in his arm weakness and gait. He regained the ability to walk without any assistance and has continued to experience progressive reduction in his residual deficits of right face numbness and paresthesia. During treatment with pembrolizumab, he did experience a mild rash, which was evaluated by dermatology and felt to be more consistent with his known history of rosacea than an immunotherapy-related rash. Repeat MRI as of November 2018 has demonstrated continued response with near complete resolution in enhancement along the pontomedullary junction in the region of CN VI, with stable disease at the right clivus, and with no new areas of enhancement (Figure 2).

## Discussion

Metastatic or unresectable cutaneous SCC is an aggressive malignancy with limited options for treatment. Currently, response rates to traditional therapies are less than 30%.^16^ This patient possessed the adverse prognostic features of a poorly differentiated subtype of SCC and symptomatic PNI. In a systematic review, clinical PNI is associated with a 2-fold increase in the risk of recurrence and 4.5 times increase in the risk of disease-specific death compared with incidental PNI found on histologic review.^3,7^ This patient received adjuvant radiation as per National Comprehensive Cancer Network guidelines^17^; however, his disease still recurred and progressed via perineural spread to the brainstem. Recent data suggest that anti-PD1 therapy with cemiplimab may be an effective and well-tolerated treatment for metastatic cutaneous SCC. However, the optimal treatment for the spindle cell variant and metastatic SCC to the brain has not been established, and the data regarding other anti-PD-1 therapies for this disease is limited.

Currently, extensive work is being done to study the predictors of anti-PD-1 antibody response. In non–small cell lung carcinoma, response to anti-PD-1 therapy in unselected patients range from 15% to 20%. Following stratification by PD-L1 expression, response rates are as high as 40%, but can be as low as 15%. However, antitumor responses have been documented in PD-L1 negative tumors as well.^18^ Thus, PD-L1 expression is not the most robust marker for anti-PD-1 antibody efficacy. Other factors that have been found to be in play include the tumor microenvironment with tumor infiltrating lymphocytes, mutational load, and DNA mismatch repair deficiency.^13^ Given the evidence that SCCs possess high mutational burdens and propagate more rapidly in immunosuppressed hosts, the role for anti-PD antibody therapy is promising.^19^ Due to the location of this patient’s SCSCC metastases, biopsy for biomarker analysis could not be performed. In the phase II cemiplimab trial, 47% of the metastatic SCC patients had a response with over half of that subgroup maintaining a response after 6 months of follow-up.^14^ Comparatively, our patient has maintained a durable response for 18 months and counting. The generalizability of cemiplimab to our patient is limited given that Migden et al do not detail the effects of cemiplimab on active brain metastases.^14^ On the other hand, data does exist for pembrolizumab’s activity in brain metastasis. In a phase II study of melanoma patients with asymptomatic 5 to 20 mm brain metastases treated with pembrolizumab, 26% of patients demonstrated either a partial or complete CNS response.^20^ Encouragingly, our patient not only experienced sustained progression-free survival when treated with pembrolizumab, but in fact showed clinical improvement from highly symptomatic CNS disease. Therefore, this case suggests that anti-PD-1 therapy may represent an effective and well-tolerated treatment for patients with metastatic SCSCC and great efficacy in controlling CNS involvement.

## Conclusions

This case suggests that anti-PD-1 therapy with pembrolizumab may represent an effective and well-tolerated treatment for patients with SCSCC with metastasis to the CNS.
